# AI companies' strategies with traditional vs. digital assets amid geopolitical and banking crises

**DOI:** 10.1016/j.heliyon.2024.e40382

**Published:** 2024-11-14

**Authors:** Wael Dammak, Mohamed Fakhfekh, Hind Alnafisah, Ahmed Jeribi

**Affiliations:** a Eslsca Business School–CERFIM, Paris, France; b Higher Institute of Business Administration of Sfax, Tunisia; c Department of Economics, College of Business Administration, Princess Nourah bint Abdulrahman University, P.O.Box 84428, Riyadh, 11671, Saudi Arabia; d Faculty of Economics and Management of Mahdia, Tunisia

**Keywords:** Intelligence artificial companies, Hedging, Safe haven, Traditional assets, Digital assets, Crises

## Abstract

The financial industry evolves rapidly, offering numerous opportunities but also introducing inherent risks that require careful navigation by institutions. This paper explores the performance of traditional and digital assets as tools for hedging, diversification, and safe-haven strategies during the Russian-Ukrainian crisis and the Silicon Valley Bank (SVB) collapse. Utilizing daily data from April 30, 2021, to September 15, 2023, and employing the Asymmetric Dynamic Conditional Correlation (ADCC) model, which combines four Generalized Autoregressive Conditional Heteroskedasticity (GARCH) family models and two residual distributions, we conduct a comprehensive analysis. The results indicate that Bitcoin's role as a safe haven is limited during the geopolitical crisis, impacting specific stocks, while gold's effectiveness varies. Digix Gold (DGX) demonstrates robust safe-haven properties, and Paxos Gold (PAXG) proves notably effective for certain stocks. Before the SVB crisis, gold primarily acts as a diversifier but later emerges as a significant safe haven during the banking crisis. These findings provide valuable insights to portfolio managers, emphasizing the importance of adaptive asset allocation in addressing financial uncertainties.

## Highlights

• AI sector is rapidly growing, attracting substantial investor interest.
• Strategies for diversifying portfolios heavily focused on AI.
• The potential of gold, Bitcoin, and gold-backed cryptocurrencies for diversification in AI-centered portfolios.
• Dynamic correlations between AI firms and alternative investments.
• PAXG and gold emerge as effective hedges, particularly during periods of uncertainty, advocating for dynamic portfolio management.

## 1 Introduction

Artificial Intelligence (AI) is ushering in a wave of new opportunities within the financial industry, marking a pivotal shift in how financial services operate. However, as these opportunities emerge, it is crucial for financial institutions to remain cognizant of the potential risks associated with employing this technology. This balance between harnessing AI's potential and managing its risks underscores the critical role AI companies play in the sector's transformation. The realm of Financial Technology (FinTech) is notably characterized by a significant integration of AI, highlighting the crucial contributions of AI entities. Their involvement does more than just revolutionize traditional financial processes; it also propels the industry toward unprecedented efficiency and innovation. This dual impact of AI—driving advancement while presenting new challenges—demonstrates the vital importance of these companies in shaping the future of finance. The KPMG report of2022^1^ reveals a striking surge in global investments in FinTech companies, reaching a record-breaking $107.8 billion in 2022. This is a significant leap from the $31 billion recorded in 2017. Such a rapid increase highlights an impressive compound annual growth rate of 28.01 % in the FinTech sector. This growth trajectory not only demonstrates the sector's vital role but also establishes it as a dominant force in financial innovation. The persistent expansion of FinTech suggests it will continue to be a significant player in shaping the financial landscape [[1], [2], [3]]; [4].

Recently, the FinTech sector has undergone a remarkable boom, primarily driven by innovative companies leveraging advanced technologies to significantly influence the financial landscape. The Financial Stability Board (2017) aptly defines FinTech as “technologically-driven financial innovation that may introduce new business models, applications, processes, or products, with a substantial impact on financial markets, institutions, and the provision of financial services.” At its heart, FinTech is about utilizing technology to revolutionize financial service delivery, as noted by Thakor [5]. This evolving sector stands at the forefront of financial innovation, reshaping the industry's landscape and its service offerings.

Recent events have significantly disrupted global financial markets, marked by challenges such as the COVID-19 pandemic, Russia's invasion of Ukraine, fluctuations in oil prices, and the collapse of SVB. These developments have led to increased uncertainty and volatility [[6], [7], [8], [9], [10], [11]] [12]; [13]; [14]. While there has been extensive research on the impact of the Russia-Ukraine conflict on financial markets [[15], [16], [17], [18], [19]], there's a notable gap in understanding its effects on the FinTech sector. The fallout from the SVB collapse has also been a subject of study [10,20,21], contributing to a broader comprehension of financial market dynamics in tumultuous times.

Consequently, the recent crises have had a clear impact on specific financial indices, such as the Global Robotics and AI Thematic Index (IGRAITI) and the Developed Market FinTech Index. These events have highlighted market inefficiencies and increased anxiety within financial systems. Notably, these crises have unfolded concurrently, adding to the complexity of the FinTech markets [22,23]. This complexity provides a unique opportunity to study the effects of recent events, such as the Russia-Ukraine conflict and the SVB collapse, on these emerging asset classes. The goal is to identify financial instruments that can act as safe havens in such situations. This research is particularly crucial for portfolio investors who depend on FinTech stocks and digital assets to mitigate risks in volatile markets.

During times of financial market volatility and uncertainty, the search for potential safe-haven assets intensifies. Gold and Bitcoin, often referred to as “digital gold,” have gained popularity due to their potential as hedges and safe assets [[24], [25], [26], [27]]. Gold, a traditional safe-haven asset, is known for its ability to retain value over time and act as a hedge against crises, inflation, and geopolitical tensions. Its role in diversifying portfolios and managing risk is vital for reducing investment risks and stabilizing performance during market instability [[28], [29], [30]]. Bitcoin, on the other hand, offers new diversification opportunities [31] and potential for arbitrage [32]. It's increasingly seen as a valuable component of diversified portfolios, complementing traditional assets like gold in mitigating risk. Additionally, the growing interest in gold-backed cryptocurrencies highlights their potential as safe-haven assets. These gold-backed digital currencies are becoming important tools for asset managers, providing a hedge against the unpredictable nature of stock market fluctuations [[33], [34], [35]].

Driven by diverse outcomes in earlier studies, our research aims to assess the hedging and safe-haven capabilities of Bitcoin, gold, and gold-backed stablecoins (PAXG and DGX) in relation to the stocks of seven leading AI companies (NVIDIA Corporation, Symbotic Inc., Helix Energy Solutions Group, Inc., C3.ai, Inc., ATS Corporation, Intuitive Surgical, Inc., and PROS Holdings, Inc.).^2^ This analysis covers the period from April 30, 2021, to September 15, 2023. We aim to explore how these different assets perform in terms of hedging and diversification for these major technology companies and AI stocks. The performance of these companies varied significantly, with NVIDIA Corporation achieving a high of 221.37 % and PROS Holdings at a lower 42.83 % (as of November 2023). Such variations in performance are likely attributed to their unique attributes, scale, and the speed at which they respond to market shocks induced by crises.

Furthermore, this research paper focuses on assessing the risk associated with certain financial assets during two significant crises: the conflict between Russia and Ukraine and the recent banking crisis. Methodologically, we employ the Asymmetric Dynamic Conditional Correlation (ADCC) model. The primary aim of this study is to explore the effects of the Ukrainian war and the banking crisis on the correlation of returns in financial series, with an emphasis on the implications for portfolio management. This involves a detailed examination of specific instruments—gold, Bitcoin, PAXG, and DGX—within an investment portfolio. Our approach utilizes the variance equation from the GARCH (Generalized Autoregressive Conditional Heteroskedasticity) model family, encompassing four distinct models: GARCH, FIGARCH (Fractionally Integrated GARCH), EGARCH (Exponential GARCH), and TGARCH (Threshold GARCH). These models are adept at capturing the nuanced characteristics often seen in financial series, such as asymmetry and the lasting effects of market shocks. To further refine our analysis, we incorporate two types of residual distributions: the Student's t-distribution and the skewed Student's t-distribution. This integration results in a suite of eight comprehensive models. The selection of these models is driven by their ability to represent the unique features commonly observed in financial data. Our study includes a comparative analysis to identify the most appropriate model for accurately describing the correlation dynamics between each pair of variables in our dataset. In this analysis, our goal is to provide valuable insights into how these financial assets behave during crises, offering useful advice to investors and portfolio managers.

This article addresses a significant gap in existing research by assessing the safe-haven properties of gold, Bitcoin, and gold-backed stablecoins relative to the stocks of the top seven AI companies. It highlights the critical role of the Russia-Ukraine crisis and the SVB collapse in this evaluation. Our selection of these leading technology and AI companies is driven by their standout performance in advanced economies. During periods of financial stress, these fintech and AI stocks exhibit unique responses, prompting an analysis of how they interact with alternative assets like Bitcoin, gold, and gold-backed stablecoins. This study, therefore, offers a nuanced exploration of asset behavior under different economic pressures, contributing valuable insights to the field of financial risk management.

Hedging the risk of AI company stocks is essential due to several factors. Firstly, while AI companies offer significant growth potential and innovation, they also operate in dynamic and rapidly evolving industries. This volatility can lead to unpredictable fluctuations in stock prices, making it crucial for investors to mitigate potential losses. Additionally, AI companies are often at the forefront of technological advancements, which can expose them to regulatory changes, cybersecurity threats, and market disruptions. By hedging their positions, investors can protect their portfolios from adverse market movements and unforeseen events, thus reducing overall risk exposure. Furthermore, hedging allows investors to maintain a balanced portfolio and diversify their holdings, mitigating the impact of any negative developments specific to the AI sector. Overall, hedging the risk of AI company stocks helps investors safeguard their investments and preserve capital in an increasingly complex and uncertain market environment.

The structure of this article is organized as follows: Section 2 commences with a comprehensive literature review. This is followed by Section 3, which offers a detailed description of the data utilized in the study. Section 4 then introduces the methodology employed in our analysis. The findings of the research, along with a thorough discussion, are presented in Section 5. Finally, the article concludes with Section 6, where we provide concluding remarks and reflections on the study.

## 2 Literature review

### 2.1 Exploring the dynamics of FinTech market

In recent decades, advancements in information and communication technologies (ICT) have revolutionized the financial sector, enhancing productivity and introducing new service approaches, particularly in developing countries [36]. This technological fusion, known as FinTech, provides numerous benefits, including cost-effective, secure, and user-friendly financial transactions [37]. The complexity of FinTech and blockchain markets represents a significant aspect of finance and economics literature. These markets offer a context for examining the effects of crises on new asset classes and guiding investment decisions, with a focus on the interconnectedness within these markets from a contagion perspective. This is crucial for understanding portfolio risk management, resource allocation, and financial instrument valuation [[38], [39], [40]].

Studies such as those by Le et al. [41] have investigated spillover effects among FinTech stocks and other financial assets during the COVID-19 crisis, highlighting increased volatility transmission. This finding is echoed in Demiralay's [42] research, which observed heightened co-movements between AI and Robotics stocks and traditional assets during periods of market turbulence. Urom [43] explored the connection between FinTech and green assets, while Rabbani et al. [44] examined the dynamic interconnections among FinTech, AI stocks, Sukuk, and Islamic equity markets during significant crises, including the Russia-Ukraine War and COVID-19.

Further research by Abakah, Adeabah et al. [45] and Alshater et al. [46] has examined the influence of public sentiment and geopolitical events, such as the Russia-Ukraine war, on FinTech and blockchain stocks. These studies indicate significant impacts on returns, volatility, and investment decisions. Similarly, Banerjee et al. [47] analyzed the relationship between traditional finance and FinTech ETFs, particularly during bank collapses, highlighting increased spillovers in returns and volatility. Motivated by these mixed findings, researchers like Manda [48] and Ali et al. [49] have investigated the crisis's impact on the interconnectedness between FinTech and traditional assets, revealing substantial changes in digital currency markets and AI stocks that affect both returns and volatility. The link between FinTech and blockchain is also crucial for understanding contagion effects, influencing resource allocation, asset pricing, and risk management. Notably, investors' behaviors, including risk tolerance and expectations, differ significantly during crises [37,38,39,50,51].

Li et al. [52] and Chaudhry, Ahmed et al. [53] have made significant contributions to the development of risk assessment methods and studies on the systemic impacts of FinTech and traditional financial firms. Their research underscores the necessity for regulatory measures in the technology sector, akin to those in the financial industry, due to evolving risk patterns and systemic effects. Driven by ICT, FinTech represents a disruptive and high-risk force within the financial sector, particularly during market crises. Consequently, FinTech stocks may not consistently provide reliable hedging opportunities against market fluctuations.

### 2.2 Hedging efficacy of gold, cryptocurrencies, and gold-backed assets in times of financial market turbulence

The recent surge in volatility across financial markets has heightened concerns regarding the potential for amplified losses during consecutive crises. As a result, investors are actively exploring novel alternative assets, such as gold, Bitcoin, and gold-backed assets, as potential hedges within their portfolios during these turbulent times [40,44,54,55]. Extensive research has been conducted on the hedging and safe-haven attributes of these assets, particularly within global equity markets [35,[56], [57], [58]].

Gold, as a precious metal, has long been a significant component of global investment portfolios, with its appeal intensified by its hedging properties amid economic uncertainties, geopolitical tensions, fluctuating currencies, inflation fears, soaring oil prices, and volatile stock markets [59]. It has been identified as a robust safe haven during the COVID-19 pandemic [[60], [61], [62], [63]]. For instance, [49] examined the role of precious metals, particularly gold, in diversifying portfolios associated with the Dow Jones Islamic (DJI) equity index during the pandemic. Their findings indicate that gold significantly reduces downside risk in these portfolios, particularly outside the Asia-Pacific region, in contrast to silver and platinum. Będowska-Sójka et al. [64] further emphasized gold's effectiveness as a hedge during geopolitical crises. Additionally, Azmi [65] highlighted gold's emergence as a safe haven during the collapse of Silicon Valley Bank.

Cryptocurrencies, particularly Bitcoin, have gained traction as potential safe-haven assets. Emerging in the aftermath of the 2008 financial crisis (Nakamoto, 2008), Bitcoin has been recognized by researchers such as Bouri et al. [66], Guo et al. [67], and Fabris & Ješić [68] for its potential as a safe haven or hedging asset. However, some studies present contrasting views; for instance, Raheem [69] concluded that Bitcoin did not demonstrate safe-haven properties during the COVID-19 pandemic, while Umar et al. [70] identified it as a significant asset during the Russian-Ukrainian conflict.

Gold-backed assets have also been scrutinized, especially during downturns like the COVID-19 pandemic. Pho et al. [71] and Díaz et al. [33] emphasized their effectiveness as portfolio tools, offering risk levels comparable to Bitcoin but lacking the safe-haven appeal associated with physical gold, as noted by Jalan [34]. Additionally, Chevallier [35] identified gold-backed tokens as viable safe assets for investment managers at the onset of the COVID-19 pandemic.

Díaz et al. [33] evaluated stablecoins, including Tether, USD Coin, and Digix Gold, highlighting their potential to mitigate downside risk in cryptocurrency portfolios. In contrast, Pho et al. [71] posited that gold serves as a more effective diversifier than cryptocurrencies for risk-averse investors. Furthermore, Wen et al. [72] established gold as a safe haven in oil and stock markets during the COVID-19 crisis. However, comparative studies, such as those by Chemkha et al. [73] and Shahzad et al. [29], are limited in assessing the roles of gold and Bitcoin across multiple markets. Fakhfekh et al. [28] underscored gold's effectiveness as a safe haven for G7 investors during the Russia-Ukraine crisis, while cryptocurrencies demonstrated mixed results as diversifying assets in G7 stock markets. Adekoya et al. [74] revealed a shift in Bitcoin's role from a shock transmitter to a receiver during the Russia-Ukraine conflict. Additionally, Jin and Tian [57] examined cryptocurrencies as safe havens amid the SVB collapse, finding that they outperformed gold in terms of return and volatility stability.

Finally, Huynh et al. [75] examined the role of AI and robotics stocks amid economic instability, highlighting the importance of Bitcoin and gold as essential hedging tools, particularly during economic downturns. This study illuminates the shifting landscape of investment strategies, where traditional assets like gold are assessed alongside emerging asset classes such as cryptocurrencies and tech stocks.

In summary, while gold, gold-backed assets, and Bitcoin have demonstrated considerable resilience during periods of instability, recent events—including the pandemic, geopolitical conflicts, and banking crises—have introduced uncertainty regarding their effectiveness as diversifiers, hedges, and safe-haven assets. This situation underscores the necessity for reevaluation, especially concerning their interactions with FinTech markets.

## 3 Data source and description

The data for all financial assets in this study were sourced from two databases: Data Stream and coincapmarket.com. The assets include indices of AI stocks, specifically NVIDIA Corporation (NVDA), Symbotic Inc. (SYM), Helix Energy Solutions Group, Inc. (HLX), C3.ai, Inc. (AI), ATS Corporation (ATS), Intuitive Surgical, Inc. (ISRG), PROS Holdings, Inc. (PRO), and the AI index. Additionally, the analysis encompasses digital and financial assets, including Bitcoin, gold, and gold-backed cryptocurrencies such as Digix Gold (DGX) and Paxos Gold (PAXG). The dataset spans from April 30, 2021, to September 15, 2023, totaling 620 observations for each selected index. This time frame was specifically chosen to capture the period of geopolitical tensions between Russia and Ukraine, as well as the banking crisis, extending up to September 15, 2023. This comprehensive data collection approach facilitates a detailed analysis of the impact of these significant events on the financial assets under study.

Daily returns are defined as $R_{t}=\mathrm{Ln}\left(P_{t}/P_{t-1}\right)\times 100$, where $P_{t}$ represents the stock's closing price on day t.

Table 1 summarizes the descriptive statistics for the study's hedging instruments and technology stocks. Among the hedging instruments analyzed, gold exhibits the highest average return at 0.014, closely followed by Paxos Gold (PAXG), which has an average return of 0.011. In contrast, Bitcoin and Digix Gold (DGX) show negative average returns of −0.12 and −0.036, respectively.

**Table 1:** Descriptive statistics.

	Mean	Std. Dev.	Skew.	Kurt.	J- Bera	Q(12)	Q^2^(12)	LM(12)
**NVDA**	0,173	3384	0,559	3191	298,272∗∗∗	9200	8314	18,137∗
**SYM**	0,179	6035	3228	61,669	99835,630∗∗∗	34,301∗∗∗	98,814∗∗∗	109,390∗∗∗
**HLX**	0,148	3543	0,361	1485	71,440∗∗∗	19,970∗	16,729	24,096∗∗
**AI**	−0,143	5375	0,160	4376	501,991∗∗∗	9376	45,919∗∗∗	37,364∗∗∗
**ATS**	0,100	2228	0,553	3661	381,476∗∗∗	20,020∗∗∗	50,385∗∗∗	55,292∗∗∗
**ISRG**	0,004	2162	−0,491	6161	1013,519∗∗∗	11,875	27,499∗∗∗	21,702∗∗
**PRO**	−0,043	3269	0,066	0,733	14,774∗∗∗	18,682∗	41,631∗∗∗	26,886∗∗∗
**AI index**	−0,049	1775	0,000	0,817	17,737∗∗∗	10,772	71,184∗∗∗	43,872∗∗∗
**Bitcoin**	−0,125	3975	−0,781	6173	1055,594∗∗∗	13,284	10,546	19,609∗
**Gold**	0,014	0,856	−0,053	1126	33,755∗∗∗	18,733∗	24,984∗∗	20,283∗
**DGX**	−0,036	13,815	2127	81,553	173158,400∗∗∗	34,338∗∗∗	19,673∗	19,937∗
**PAXG**	0,011	0,857	0,100	1334	47,894∗∗∗	9136	63,549∗∗∗	38,506∗∗∗

Notes: SD represents the standard deviation; J. Bera refers to the Jarque-Bera normality test. The symbols ∗, ∗∗, and ∗∗∗ denote significance levels at 10 %, 5 %, and 1 %, respectively.

Turning to the technology and AI stocks, NVIDIA Corporation (NVDA), Symbotic Inc. (SYM), Helix Energy Solutions Group, Inc. (HLX), ATS Corporation (ATS), and Intuitive Surgical, Inc. (ISRG) all report positive mean returns. Conversely, AI, PROS Holdings, Inc. (PRO), and the AI Index are characterized by negative mean returns. Notably, DGX displays a high level of volatility, with a standard deviation of 13.815, indicating a greater degree of price fluctuation compared to the other assets studied.

The skewness coefficients in our analysis reveal that only Intuitive Surgical, Inc. (ISRG), Bitcoin, and gold exhibit left-skewed distributions, as evidenced by their negative values. In contrast, the remaining assets studied demonstrate a positive skew, indicating rightward lean in their return distributions. Moreover, all return series show pronounced leptokurtosis, suggesting that they have more pronounced peaks and heavier tails compared to a normal distribution. This observation is further supported by the results of the Jarque-Bera test, which reject the null hypothesis of normality for the return series. This rejection highlights the significant departure of the return series from normality, emphasizing the necessity of accounting for these non-normal characteristics when estimating the DCC-GARCH models. Incorporating these aspects into our modeling approach is essential to ensure a more accurate and representative analysis of the financial data.

The results from the Ljung-Box test indicate the presence of autocorrelation in the majority of the return and squared return series, contradicting the initial assumption of their absence. This finding suggests that volatility clustering is likely present in most of the return datasets. The application of the GARCH model configuration effectively addresses this issue. Furthermore, the Lagrange multiplier test confirms the persistent presence of an ARCH effect across all return datasets, reinforcing the justification for employing the ARCH model to accurately capture the volatility clustering observed in the data.

**Fig. 1:**
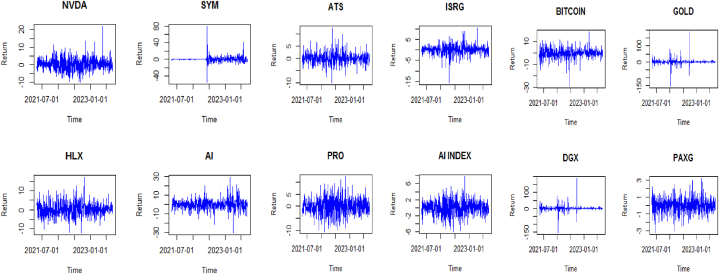
Return evolution series.

Fig. 1 illustrates the return trends for all the series under study. Prior to the onset of the Russia-Ukraine conflict, the datasets display a combination of upward and downward movements. However, the emergence of the war, coupled with the subsequent banking crisis, signals a significant shift, with most return datasets experiencing substantial declines. Notably, gold and the gold-backed digital currency DGX stand out as exceptions to this trend. During both crises, these assets exhibit signs of stability, contrasting sharply with the overall downward trajectory observed in the other datasets.

## 4 Econometric methodology

This research paper investigates the risks associated with selected financial assets during two significant crises: the Russia-Ukraine war and the recent banking crisis. The study encompasses seven major technology and AI stocks—NVIDIA (NVDA), Symbotic (SYM), Helix Energy Solutions Group, Inc. (HLX), C3.ai, Inc. (AI), ATS Corporation (ATS), Intuitive Surgical, Inc. (ISRG), PROS Holdings, Inc. (PRO), and the AI INDEX—alongside hedging instruments such as Bitcoin, Gold, and gold-backed stablecoins (PAXG and DGX).

The primary objective is to explore how these crises influence the correlation of financial returns and their implications for portfolio management. To achieve this, we employ the Asymmetric Dynamic Conditional Correlation (ADCC) model, utilizing variance equations from four models within the GARCH family: GARCH, FIGARCH, EGARCH, and TGARCH. Our approach also incorporates two residual distributions: the Student's t-distribution and the Skewed Student's t-distribution, resulting in a total of eight different models.

The selection of these models is guided by typical characteristics observed in financial series, including asymmetry and the persistent effects of shocks. Our aim includes conducting a comparative analysis to identify the most suitable model for elucidating the correlation dynamics between the various pairs of variables in our dataset.

### 4.1 The GARCH model

The standard GARCH model developed by Bollerslev [76] can be written as follows:

(1) $$\begin{array}{c} \sigma_{t}^{2}=\omega +\sum_{j=1}^{q}\alpha_{j}\varepsilon_{t-j}^{2}+\sum_{j=1}^{p}\beta_{j}\sigma_{t-j}^{2} \end{array}$$

where, $\sigma_{t}^{2}$ denotes the conditional variance, with ω representing the intercept, and $\varepsilon_{t}^{2}$ as the residuals from the mean filtering process. The GARCH order is defined by (q, p) (ARCH, GARCH).

### 4.2 The EGARCH model

The exponential model, as formulated by Ref. [77], is defined in the following manner:

(2) $$\begin{array}{c} \log\left(\sigma_{t}^{2}\right)=\omega +\sum_{j=1}^{q}\left(\alpha_{j}z_{t-j}+\gamma_{j}\left(\left|z_{t-j}\right|-E\left|z_{t-j}\right|\right)\right)+\sum_{j=1}^{p}\beta_{j}\;\log\left(\sigma_{t-j}^{2}\right) \end{array}$$

In this model, the coefficient $\alpha_{j}$ captures the sign effect, while $\gamma_{j}$ represents the size effect.

The expected value of the absolute standardized innovation, denoted as, $z_{t}$, is:

(3) $$\begin{array}{c} E\left|z_{j}\right|=\int_{-\infty }^{\infty }\left|z\right|f\left(z,0,1,\ldots \right)dz \end{array}$$

The estimated persistence $\hat{P}$ is given by: $\hat{P}=\sum_{j=1}^{p}\beta_{j}$.

### 4.3 The FIGARCH model

Inspired by advancements in long-memory processes, especially ARFIMA-type models, Baillie et al. [78] proposed the Fractionally Integrated Generalized Autoregressive Conditional Heteroscedasticity (FIGARCH) model. This model is specifically designed to capture long memory in financial time series, characterized by a hyperbolic decay pattern of shocks. Unlike the standard GARCH model, where shocks fade away exponentially, or the Integrated GARCH model that assumes indefinite persistence of shocks, the FIGARCH model distinguishes itself by how shocks diminish at a hyperbolically slower rate. The formulation of this model is as follows:

(4) $$\begin{array}{c} \phi \left(L\right){\left(1\;–\;L\right)}^{d}\varepsilon_{t}^{2}=\omega +\left[1-\beta \;\left(L\right)\right]\upsilon_{t} \end{array}$$

where, the term ${\left(1\;–L\right)}^{d}$ represents the fractional differencing operator, with ∈[0,1]. This model seamlessly transitions to a GARCH(p, q) process when d=0 and to an IGARCH(p, q) process when d=1, thereby incorporating these scenarios as specific instances within its broader framework. Essentially, the FIGARCH model is characterized by its flexibility, adeptly representing long memory effects in time series data. This adaptability makes it particularly well-suited for achieving the objectives outlined in this study.

### 4.4 The TGARCH model

Another variant of the GARCH model, specifically designed to model leverage effects, is the Threshold GARCH (TGARCH) model. Its formulation is presented as follows

(5) $$\begin{array}{c} \sigma_{t}^{2}=a_{0}+\sum_{i=1}^{P}a_{i}\varepsilon_{t-i}^{2}+\sum_{i=1}^{P}\gamma_{i}S_{t-i}\varepsilon_{t-i}^{2}+\sum_{j=1}^{q}b_{j}\sigma_{t-j}^{2} \end{array}$$

$$\text{where},S_{t-i}=\left\{ \begin{array}{c} 1\;if\varepsilon_{t-i}\langle 0\;<\\ 0\;if\varepsilon_{t-i}\geq 0 \end{array}\right.$$

In other words, depending on whether $\varepsilon_{t-i}$ is greater or less than the threshold value of zero, $\varepsilon_{t-i}^{2}$ has different effects on the conditional variance $\sigma_{t}^{2}$: when $\varepsilon_{t-i}$ is positive, the total effects are given by $a_{i}\varepsilon_{t-i}^{2}$; when $\varepsilon_{t-i}$ is negative, the total effects are given by $\left(a_{i}+\gamma_{i}\right)\varepsilon_{t-i}^{2}$. Therefore, one might expect $\gamma_{i}$ to be positive so that bad news has a more significant impact.

### 4.5 The ADCC model

To address the potential asymmetry in the underlying correlation structure, we implement the ADCC model, as introduced by Cappiello et al. [79]. Additionally, we follow the two-step estimation approach described by Engle and Sheppard [80]. In the first step, we estimate the mean and GARCH parameters. In the second step, we focus on estimating the dynamic conditional correlations. The ADCC model, specifically designed for these conditional dynamic correlations, is presented in its general form as follows:

(6) $$\begin{array}{c} r_{t}=\mu +\varphi r_{t-1}+\varepsilon_{t} \end{array}$$

(7) $$\varepsilon_{t}|I_{t-1}∼t\left(0,H_{t},v\right)$$

(8) $$\begin{array}{c} H_{t}=D_{t}R_{t}D_{t} \end{array}$$

(9) $$\begin{array}{c} D_{t}=diag\left(h_{1,t}^{\frac{1}{2}},\ldots h_{n,t}^{\frac{1}{2}}\right) \end{array}$$

(10) $$\begin{array}{c} R_{t}={diag\left(Q_{t}\right)}^{-\frac{1}{2}}Q_{t}{diag\left(Q_{t}\right)}^{-\frac{1}{2}} \end{array}$$

where, $R_{t}$ is the (n×1) vector of the returns series.

Eq. (6) corresponds to the mean equation where μ and φ are the two parameters to be estimated, and $\varepsilon_{t}$ is the vector of the residuals as defined in Eq. (7). $H_{t}$ is (n×n) the conditional covariance matrix and $z_{t}$ is an (n×1) random vector that have a mean 0 vector and the identity matrix as a variance. $D_{t}$ is the diagonal matrix, with the i-th diagonal element, $h_{i,t}^{1/2}$ for i=1,…,n, is the conditional standard deviation of the i-th cryptcurrency. These $h_{i,t}^{1/2}$ are obtained from the first step of the estimation of the GARCH model.

Finally, $R_{t}=\left\{\rho_{ij,t}\right\}$ is the matrix of correlation with ones on the diagonal and all other elements smaller than 1 in absolute value. The dynamic of $Q_{t}$ in Eq. (10) is given by:

(11) $$\begin{array}{c} Q_{t}=\left(\bar{R}-a\bar{R}-b\bar{R}-g\bar{N}\right)+az_{t-1}z_{t-1}^{\prime }+bQ_{t-1}+gn_{t-1}n_{t-1}^{\prime } \end{array}$$

where, a, b, and g are positive scalar parameters. $z_{\text{it}}=\left(\varepsilon_{\text{it}}/\sqrt{h_{\text{it}}}\right)$ is the vector of standardized residuals $.\;\bar{R}=E\left[z_{t}z_{t}^{\prime }\right]$, $\bar{N}=E\left[n_{t}n_{t}^{\prime }\right]$, The $\bar{R}$ and $\bar{N}$ are estimated using their sample analogues given by $T^{-1}\sum_{t=1}^{T}z_{t}z_{t}^{\prime }$ and $T^{-1}\sum_{t=1}^{T}n_{t}n_{t}^{\prime }$, respectively (see Cappiello et al.[79] for more details).

### 4.6 Hedging, diversifying and safe haven properties

After estimating the ADCC/GARCH model, we extract time-varying correlations, denoted as $\rho_{t}$, from the model 10. These $\rho_{t}$ values are then regressed against dummy variables that represent periods of market turbulence. This regression analysis is conducted to test the efficacy of various instruments as hedges and safe-haven assets against the risks associated with technology company stocks:

(12) $$\begin{array}{c} \rho_{t}=\gamma_{0}+\gamma_{1}D\left(R_{asset}q_{10}\right)+\gamma_{2}D\left(R_{asset}q_{5}\right)+\gamma_{3}D\left(R_{asset}q_{1}\right) \end{array}$$

where, D represents dummy variables that capture extreme movements in the underlying stock returns at the 10 %, 5 %, and 1 % quantiles of the most negative stock returns. The instruments (Bitcoin, Gold, PAXG, and DGX) provide weak hedging if $\gamma_{0}$ is zero, or strong hedging if $\gamma_{0}$ is negative. The instruments in question act as a weak safehaven if the coefficients $\gamma_{1}$, $\gamma_{2}$, or $\gamma_{3}$ are slightly different from zero, or a strong safehaven if they are negative.

To assess the instruments as hedges and safe-haven assets against the risk of the stock market during a crisis, an adapted version of a dummy variable regression model used by Baur and McDermott [81] and Ali et al. [49] is empirically tested as follows:

(13) $$\begin{array}{c} \rho_{t}=\beta_{0}+\beta_{1}D\left(crisis\right) \end{array}$$

where, a dummy variable is set to one during the crisis period. We estimated this model twice, with the first model considering the war between Russia and Ukraine starting on February 11, 2022 and continuing for 28 trading days, and the second model considering the recent US financial banking crisis starting on March 07, 2023 and continuing for 20 trading days. The instruments serve as weak hedges if $\beta_{0}$ is statistically insignificant from zero, or strong hedges if $\beta_{0}$ is negative for different sectors. The instruments act as weak safe havens if $\beta_{1}$ is statistically non-significant compared to zero, or strong safe havens if $\beta_{1}$ is negative for different sectors.

## 5 Empirical results and discussion

### 5.1 ADCC characteristics

Before estimating the various GARCH/ADCC models, we first determined the best model to explain the correlation dynamics. This selection was made from four models within the GARCH family: GARCH, FIGARCH, EGARCH, and TGARCH, combined with ADCC under two residual distributions, namely Student and Skewed Student distributions, resulting in a total of eight models. The preferred model was chosen based on minimizing information criteria. The findings indicated that for Bitcoin, the optimal models for examining the conditional dynamic correlation with different stocks are as follows: GARCH/ADCC, EGARCH/ADCC, and TGARCH/ADCC are best suited for pairs with NVDA, HLX, PRO, and AI index; AI, ATS, and ISRG; and SYM, respectively. In the case of gold, the TGARCH/ADCC model emerged as the most effective for SYM, ATS, PRO, ISRG, and AI index. For DGX, the FIGARCH/ADCC model was identified as the best fit across all series. Lastly, for PAXG, the TGARCH/ADCC model is best suited for modeling its relationship with SYM, ATS, ISRG, and PRO.

Our in-depth analysis of the dynamic correlations between various financial assets through model selection has yielded the following insights.

#### 5.1.1 Correlations with Bitcoin

• For NVDA, HLX, PRO, and the AI Index, the GARCH/ADCC model proved most effective. This suggests that Bitcoin's volatility and its relationship with these tech stocks are well-represented by the GARCH model, known for handling time-varying volatility.
• The EGARCH/ADCC model was the optimal choice for examining Bitcoin's correlation with AI, ATS, and ISRG. This implies that these correlations might involve asymmetries or leverage effects, which are well-captured by the EGARCH model.
• In the case of Bitcoin's interaction with SYM, the TGARCH/ADCC model was the best fit, indicating a differential impact of positive and negative shocks on Bitcoin's correlation with SYM, a characteristic the TGARCH model is adept at modeling.

#### 5.1.2 Correlations with gold

• The TGARCH/ADCC model stood out as the most suitable for gold in its correlations with SYM, ATS, PRO, ISRG, and the AI Index. This implies that gold's relationship with these stocks is likely characterized by asymmetric reactions to market shocks, with negative movements having a greater impact than positive ones.

#### 5.1.3 Correlations with DGX

• The FIGARCH/ADCC model was the top choice across all series for DGX. This indicates that the correlation between DGX and the stocks under study is influenced by long-memory effects, where past volatility and shocks exert a lingering impact.

#### 5.1.4 Correlations with PAXG

• For PAXG's relationships with SYM, ATS, ISRG, and PRO, the TGARCH/ADCC model was found to be most fitting. This aligns with the gold findings, suggesting an asymmetric impact of market events on PAXG's correlation with these stocks.

These findings provide a comprehensive view of the intricate and diverse correlation patterns between different asset types, including traditional assets like gold and newer ones like Bitcoin and gold-backed digital currencies. The selection of specific GARCH/ADCC models for each asset pairing highlights the unique aspects of each correlation, whether it be response asymmetry to market fluctuations, long-term memory, or varying impacts of leverage. This understanding is vital for investors and portfolio managers to optimize asset allocation, enhance risk management, and refine hedging strategies, considering varying market conditions. The detailed findings from the multivariate GARCH-ADCC analysis are presented in Table 2a, Table 2b, Table 2c, Table 2da–2d. In Table 2a, the relationship between Bitcoin and various technology stocks is analyzed using the GARCH/ADCC model. One notable result is the significant first-order autoregressive component for ATS, demonstrating a particularly strong relationship at the 1 % level of significance.

**Table 2a:** Estimation results of ADCC-GARCH model for BITCOIN.^1^.

Models	GARCH/ADCC	TGARCH/ADCC	GARCH/ADCC	EGARCH/ADCC	EGARCH/ADCC	EGARCH/ADCC	GARCH/ADCC	GARCH/ADCC
Pairs	BITCOIN/NVDA	BITCOIN/SYM	BITCOIN/HLX	BITCOIN/AI	BITCOIN/ATS	BITCOIN/ISRG	BITCOIN/PRO	BITCOIN/AI INDEX
mu	−0,0358	−0,0541	−0,0358	−0,0424	−0,0424	−0,0424	−0,0358	−0,0358
ar1	−0,0312	−0,0314	−0,0312	−0,0285	−0,0285	−0,0285	−0,0312	−0,0312
omega	0,0255	0,0403	0,0255	−0,0028	−0,0028	−0,0028	0,0255	0,0255
alpha1	0,0325	0,0501∗∗	0,0325	0,0047	0,0047	0,0047	0,0325	0,0325
beta1	0,9665∗∗∗	0,9403∗∗∗	0,9665∗∗∗	0,8531∗∗∗	0,8531∗∗∗	0,8531∗∗∗	0,9665∗∗∗	0,9665∗∗∗
eta11	–	0,0913∗	–		–	–	–	–
gamma1	–	–	–	0,0829∗∗∗	0,0829∗∗∗	0,0829∗∗∗	–	–
shape	3,2719∗∗∗	2,9605∗∗∗	3,2719∗∗∗	3,1398∗∗∗	3,1398∗∗∗	3,1398∗∗∗	3,2719∗∗∗	3,2719∗∗∗
mu	0,1791	−0,0119	0,1326	−0,08	0,0653∗∗∗	0,1096	−0,0322	0,0062
ar1	0,0426	−0,0572	0,0479	0,0196	0,1459∗∗∗	0,0143	0,0052	0,0641
omega	0,0741	0,0099∗	0,0001	0,1772∗∗∗	0,6884∗	0,0197∗∗∗	0,0492	0,0225
alpha1	0,0353∗∗∗	0,0805∗∗∗	0,0264∗∗∗	0,0237∗∗∗	0,0262	0,0017∗∗∗	0,0386∗∗∗	0,0495∗∗∗
beta1	0,9599∗∗∗	0,8239∗∗∗	0,9726∗∗∗	0,9450∗∗∗	0,6007∗∗∗	0,9847∗∗∗	0,9572∗∗∗	0,9432∗∗∗
eta11	–	0,136∗∗	–	–	–	–	–	–
gamma1	–	–	–	0,1604∗∗∗	0,5307∗∗∗	0,0623∗∗∗	–	–
shape	5,3782∗∗∗	2,9066∗∗∗	6,4427∗∗∗	4,6453∗∗∗	3,2142∗∗∗	3,6985∗∗∗	14,5463∗∗	33,5029
[Joint]dcca1	0,007	0,0001	0,0061	0,0155	0	0,0162	0,0199∗	0,0176∗∗
[Joint]dccb1	0,9779∗∗∗	0,9756∗∗∗	0,9902∗∗∗	0,9745∗∗∗	0,9994∗∗∗	0,9738∗∗∗	0,9691∗∗∗	0,9762∗∗∗
[Joint]dccg1	0,0138	0,0299	0,0004	0,0001	0,0013	0,0135	0	0
[Joint]mshape	4,3945∗∗∗	4,0000∗∗∗	4,6739∗∗∗	4,1559∗∗∗	4,0000∗∗∗	4,0000∗∗∗	5,3786∗∗∗	5,6127∗∗∗

Notes: In the model, ‘mu’ represents the constant, and ‘ar(1)' denotes the AR(1) term in the mean equation. ‘omega’ is the constant in the variance equation. The terms ‘alpha1’ and ‘beta1’ correspond to the ARCH and GARCH components, respectively. ‘etat11’ is indicative of the asymmetry term, while ‘shape’ signifies the shape term in the model. Significance levels are denoted by ∗∗∗, ∗∗, and ∗, corresponding to 1 %, 5 %, and 10 %, respectively.

1 The selection of the best model was based on information criteria (we chose 4 GARCH models, namely GARCH, TGARCH, EGARCH, FIGARCH, under 2 distributions, std and sstd).

**Table 2b:** Estimation results of ADCC-GARCH model for GOLD.

Model	EGARCH/ADCC	TGARCH/ADCC	GARCH/ADCC	EGARCH/ADCC	TGARCH/ADCC	TGARCH/ADCC	TGARCH/ADCC	TGARCH/ADCC
Pairs	GOLD/NVDA	GOLD/SYM	GOLD/HLX	GOLD/AI	GOLD/ATS	GOLD/ISRG	GOLD/PRO	GOLD/AI INDEX
mu	0,0256	0,0276	0,0181	0,0256	0,0276	0,0276	0,0276	0,0276
ar1	0,0221	0,0224	0,0103	0,0221	0,0224	0,0224	0,0224	0,0224
omega	−0,017	0,049	0,0115	−0,017	0,049	0,049	0,049	0,049
alpha1	0,0588	0,06	0,0395∗∗	0,0588	0,06	0,06	0,06	0,06
beta1	0,9435∗∗∗	0,8990∗∗∗	0,9466∗∗∗	0,9435∗∗∗	0,8990∗∗∗	0,8990∗∗∗	0,8990∗∗∗	0,8990∗∗∗
eta11	–	−0,5312	–	–	−0,5312	−0,5312	−0,5312	−0,5312
gamma1	0,1196	–	–	0,1196	–	–	–	–
shape	5,8058∗∗∗	5,8243∗∗∗	5,3893∗∗∗	5,8058∗∗∗	5,8243∗∗∗	5,8243∗∗∗	5,8243∗∗∗	5,8243∗∗∗
mu	0,0862	−0,0119	0,1326	−0,08	0,0984	0,1152∗	−0,0882	−0,0141
ar1	0,038	−0,0572	0,0479	0,0196∗∗∗	0,0946∗∗	0,0089	−0,0038	0,0624
omega	4,5342∗∗∗	0,0099∗	0,0001	0,1772∗∗∗	0,0504∗∗	0,0269∗∗	0,0124	0,0157∗∗
alpha1	0,0576	0,2805∗∗∗	0,0264∗∗∗	0,1237∗∗∗	0,0302∗∗∗	0,0404∗∗∗	0,0247∗∗∗	0,0231∗∗∗
beta1	−0,8606∗∗∗	0,8239∗∗∗	0,9726∗∗∗	0,9449∗∗∗	0,9581∗∗∗	0,9575∗∗∗	0,9773∗∗∗	0,9723∗∗∗
eta11	–	0,136	–	–	1,0000∗	0,6482∗∗∗	0,9884∗∗	1,0000∗∗
gamma1	−0,0785	–	–	0,1604∗∗∗	–	–	–	–
shape	5,5674∗∗∗	2,9066∗∗∗	6,4427∗∗∗	4,6458∗∗∗	3,5294∗∗∗	4,0618∗∗∗	18,4681	99,9995
[Joint]dcca1	0,0413∗∗∗	0,0133	0,0372	0,0284∗∗∗	0,0518∗	0,0427∗∗∗	0,0375∗∗∗	0,0510∗∗∗
[Joint]dccb1	0,9348∗∗∗	0,8781∗∗∗	0,8721∗∗∗	0,9562∗∗∗	0,8870∗∗∗	0,9333∗∗∗	0,9438∗∗∗	0,9336∗∗∗
[Joint]dccg1	0,0012	0,0654	0,0001	0,0048	0,0269	0,0012	0,0016	0,0017
[Joint]mshape	6,2652∗∗∗	4,1967∗∗∗	6,2706∗∗∗	5,2330∗∗∗	5,0959∗∗∗	5,1549∗∗∗	9,9542∗∗∗	11,3609∗∗∗

Notes: In the model, ‘mu’ is used to denote the constant, and ‘ar(1)' indicates the AR(1) term within the mean equation. ‘omega’ represents the constant in the variance equation. The term ‘alpha1’ pertains to the ARCH component, and ‘beta1’ is associated with the GARCH component. ‘etat11’ is used to describe the asymmetry term, whereas ‘shape’ denotes the shape term in the model. The levels of significance are marked by ∗∗∗, ∗∗, and ∗, which correspond to 1 %, 5 %, and 10 %, respectively.

**Table 2c:** Estimation results of ADCC-GARCH model for DGX.

Model	FIGARCH/ADCC							
Pairs	DGX/NVDA	DGX/SYM	DGX/HLX	DGX/AI	DGX/ATS	DGX/ISRG	DGX/PRO	DGX/AI INDEX
mu	−0,1132	−0,1132	−0,1132	−0,1132	−0,1132	−0,1132	−0,1132	−0,1132
ar1	−0,0388	−0,0388	−0,0388	−0,0388	−0,0388	−0,0388	−0,0388	−0,0388
omega	3823	3823	3823	3823	3823	3823	3823	3823
alpha1	0,2905	0,2905	0,2905	0,2905	0,2905	0,2905	0,2905	0,2905
beta1	0,8893∗∗∗	0,8893∗∗∗	0,8893∗∗∗	0,8893∗∗∗	0,8893∗∗∗	0,8893∗∗∗	0,8893∗∗∗	0,8893∗∗∗
delta	0,9436∗	0,9436∗	0,9436∗	0,9436∗	0,9436∗	0,9436∗	0,9436∗	0,9436∗
shape	2,1373∗∗∗	2,1373∗∗∗	2,1373∗∗∗	2,1373∗∗∗	2,1373∗∗∗	2,1373∗∗∗	2,1373∗∗∗	2,1373∗∗∗
mu	0,1721	−0,0043	0,1384	−0,1791	0,0729	0,1233∗	−0,025	0,0049
ar1	0,0468	−0,0829∗	0,0515	0,0281	0,1510∗∗∗	0,0192	0,0052	0,0739∗
omega	0,0512	0,0057∗∗∗	0,0001	0,2577	0,1050∗	0,0354	0,0221	0,0144
alpha1	0,0001	0,0857	0	0,1076	0,7639∗∗∗	0,0444	0,0655	0,1326
beta1	0,9504∗∗∗	0,7787∗∗∗	0,9664∗∗∗	0,9430∗∗∗	0,9344∗∗∗	0,9550∗∗∗	0,9393∗∗∗	0,8143∗∗∗
delta	0,9504∗∗∗	0,4638∗∗∗	0,9694∗∗	0,6483∗	0,5102	0,6482∗∗∗	0,8968∗∗∗	0,6816∗∗
shape	5,0733∗∗∗	3,8162∗∗∗	6084	4,0020∗∗∗	3,2210∗∗∗	3,6259∗∗∗	13,6731	37,4384
[Joint]dcca1	0	0	0,0001	0	0	0	0	0
[Joint]dccb1	0,9644∗∗∗	0,9882∗∗∗	0,9184∗∗∗	0,9942∗∗∗	0,4009	0,6340∗∗∗	0,1542	0,8219∗∗∗
[Joint]dccg1	0,0476	0,0235	0,0001	0,0114	0,1319	0,2294	0,4948	0,135
[Joint]mshape	4,0000∗∗∗	4,0000∗∗∗	4,0000∗∗∗	4,0000∗∗∗	4,0000∗∗∗	4,0000∗∗∗	4	4,0000∗∗∗

Notes: In this model, ‘mu’ signifies the constant, and ‘ar(1)' represents the AR(1) term within the mean equation. ‘omega’ is the constant in the variance equation. The term ‘alpha1’ is associated with the ARCH component, and ‘beta1’ denotes the GARCH component. ‘etat11’ indicates the asymmetry term, while ‘shape’ refers to the shape term in the model. Significance levels are marked as ∗∗∗, ∗∗, and ∗, corresponding to 1 %, 5 %, and 10 % levels of significance, respectively.

**Table 2d:** Estimation results of ADCC-GARCH model for PAXG.

Model	EGARCH/ADCC	TGARCH/ADCC	GARCH/ADCC	EGARCH/ADCC	TGARCH/ADCC	TGARCH/ADCC	TGARCH/ADCC	GARCH/ADCC
Pairs	PAXG/NVDA	PAXG/SYM	PAXG/HLX	PAXG/AI	PAXG/ATS	PAXG/ISRG	PAXG/PRO	PAXG/AI INDEX
mu	0,011	0,0121	0,0024	0,011	0,0121	0,0121	0,0121	0,0024
ar1	−0,0173	−0,02	−0,0284	−0,0173	−0,02	−0,02	−0,02	−0,0284
omega	−0,0337	0,1288	0,113	−0,0337	0,1288	0,1288	0,1288	0,113
alpha1	0,0693∗	0,1062∗∗	0,1115∗	0,0693∗	0,1062∗∗	0,1062∗∗	0,1062∗∗	0,1115∗
beta1	0,9063∗∗∗	0,7682∗∗∗	0,7352∗∗∗	0,9063∗∗∗	0,7682∗∗∗	0,7682∗∗∗	0,7682∗∗∗	0,7352∗∗∗
eta11	–	−0,4219∗	–	–	−0,4219∗	−0,4219∗	−0,4219∗	–
gamma1	0,1704∗	–	–	0,1704∗	–	–	–	–
shape	6,5251∗∗∗	6,4893∗∗∗	6,5087∗∗∗	6,5251∗∗∗	6,4893∗∗∗	6,4893∗∗∗	6,4893∗∗∗	6,5087∗∗∗
mu	0,1141	−0,0119	0,1326	−0,08	0,0984	0,1152∗	−0,0882	0,0062
ar1	0,0458	−0,0572	0,0479	0,0196∗∗∗	0,0946∗∗	0,0089	−0,0038	0,0641∗
omega	0,0472∗∗∗	0,0099∗	0,0001	0,1771∗∗∗	0,0504∗∗	0,0269∗∗	0,0124	0,0225
alpha1	−0,0629∗∗∗	0,2805∗∗∗	0,0264∗∗∗	0,1237∗∗∗	0,0302∗∗∗	0,0404∗∗∗	0,0247∗∗∗	0,0495∗∗∗
beta1	0,9808∗∗∗	0,8239∗∗∗	0,9726∗∗∗	0,9450∗∗∗	0,9581∗∗∗	0,9575∗∗∗	0,9773∗∗∗	0,9432∗∗∗
eta11	–	0,136	–	–	0,6481∗	0,5628∗∗∗	0,9884∗∗	–
gamma1	0,0968∗∗∗	–	–	0,1604∗∗∗	–	–	–	–
shape	5,8579∗∗∗	2,9066∗∗∗	6,4427∗∗∗	4,6460∗∗∗	3,5294∗∗∗	4,0618∗∗∗	18,4681	33,5029
[Joint]dcca1	0,0429∗∗∗	0,0148	0,0261	0,0274∗∗∗	0,0555∗∗∗	0,0387∗∗∗	0,0371∗∗∗	0,0475∗∗∗
[Joint]dccb1	0,9362∗∗∗	0,9041∗∗∗	0,9343∗∗∗	0,9563∗∗∗	0,8861∗∗∗	0,9315∗∗∗	0,9438∗∗∗	0,9379∗∗∗
[Joint]dccg1	0,0027	0,0339	0,0001	0,0087	0,0151	0,012	0,0013	0,0042
[Joint]mshape	6,4623∗∗∗	4,4035∗∗∗	7,3094∗∗∗	5,2257∗∗∗	5,4806∗∗∗	5,2371∗∗∗	10,3290∗∗∗	10,4889∗∗∗

Notes: In the model, ‘mu’ denotes the constant and ‘ar(1)' indicates the AR(1) term in the mean equation. The term ‘omega’ is the constant within the variance equation. ‘alpha1’ corresponds to the ARCH term and ‘beta1’ to the GARCH term. The asymmetry term is represented by ‘etat11’, while ‘shape’ signifies the shape term in the model. Significance levels are indicated as ∗∗∗, ∗∗, and ∗, corresponding to 1 %, 5 %, and 10 %, respectively.

We also observe that the volatility of all the series' prices is persistent, as the sum of $\alpha_{1}+\beta$ is very close to one, implying that the persistence of volatility increased during the crisis and indicates a long memory process. This suggests that the effects of past market fluctuations continue to influence these financial series, a phenomenon that becomes more pronounced during times of crisis, pointing to a long memory process in the data. The AR(1)-TGARCH(1,1) model applied to Bitcoin and SYM reveals significant positive values for the “eta11” parameter, which measures asymmetry in variance. This indicates that the market's volatility has a heightened reaction to negative events compared to positive ones, a factor that can significantly impact risk management strategies. In the case of AI, ATS, and ISRG, the AR(1)-EGARCH(1,1) model shows that the “gamma1” parameter, associated with the leverage effect, is significantly positive at the 1 % level. This finding implies that negative returns tend to have a more pronounced effect on future volatility for these stocks, highlighting their sensitivity to negative market movements. Overall, these insights into the volatility and correlation dynamics between Bitcoin and various technological stocks are essential for informed investment decisions, particularly under uncertain market conditions and financial instability.

### 5.2 Diversifying, hedging, and safe haven results during the Russia-Ukraine war

Applying the methodology developed by Baur and Lucey [82], we analyzed the regression model (Eq. (12)) and detailed the results in Table 3. The analysis shows that the coefficient $\gamma_{0}$ is significantly positive across all assets studied, indicating that the potential of Bitcoin, gold, DGX, and PAXG can play the role of diversifiers before the onset of the crises in question.

**Table 3:** Estimation results of Baur and Lucey Equation.

	Coef.	Bitcoin	Role	Gold	Role	DGX	Role	PAXG	Role
**NVDA**	γ_0_	0,3875∗∗∗	VRFA	0,0756∗∗∗	DIV+ VRFA	0,1018∗∗∗	VRFA	0,0966∗∗∗	DIV+ VRFA
	γ_1_	0,0005		0,0216		0,0007		0,0045	
	γ_2_	−0,0051		−0,0147		0,0000		0,0012	
	γ_3_	0,0017		0,0154		−0,0013		0,0254	
**SYM**	γ_0_	0,0737∗∗∗	DIV+ VRFA	0,0550∗∗∗	DIV+ VRFA	0,0220∗∗∗	DIV+ VRFO	0,0369∗∗∗	DIV+ VRFA
	γ_1_	0,0001		0,0006		−0,0005∗∗		0,0003	
	γ_2_	0,0012		−0,0018		−0,0013∗∗∗		−0,0035	
	γ_3_	0,0002		0,0030		0,0017∗∗∗		0,0037	
**HLX**	γ_0_	0,1836∗∗∗	VRFA	0,1644∗∗∗	VRFA	0,0645∗∗∗	DIV+ VRFA	0,1441∗∗∗	VRFO
	γ_1_	−0,0007		0,0061		0,0001		0,0117	
	γ_2_	−0,0016		0,0087		0,0001		0,0058	
	γ_3_	0,0020		−0,0205		0,0001		−0,0277∗	
**AI**	γ_0_	0,2769∗∗∗	VRFA	0,0666∗∗∗	DIV+ VRFA	0,1238∗∗∗	VRFA	0,0607∗∗∗	VRFA
	γ_1_	0,0023		0,0279		0,0001		0,0094	
	γ_2_	−0,0043		0,0095		−0,0017		0,0198	
	γ_3_	0,0008		−0,0034		0,0012		0,0034	
**ATS**	γ_0_	0,2758∗∗∗	VRFA	0,1516∗∗∗	VRFA	0,0849∗∗∗	DIV+ VRFA	0,1401∗∗∗	VRFA
	γ_1_	−0,0008		0,0333		0,0001		0,0336	
	γ_2_	0,0004		0,0181		−0,0005		−0,0050	
	γ_3_	0,0011∗		−0,0060		0,0005		0,0136	
**ISRG**	γ_0_	0,2837∗∗∗	VRFA	0,1157∗∗∗	VRFA	0,0837∗∗∗	DIV+ VRFA	0,1047∗∗∗	VRFA
	γ_1_	0,0012		0,0209		0,0012		−0,0048	
	γ_2_	−0,0088		0,0220		0,0015		0,0138	
	γ_3_	0,0031		−0,0027		−0,0029		0,0081	
**PRO**	γ_0_	0,2368∗∗∗	VRFO	0,0529∗∗∗	DIV+ VRFA	0,0835∗∗∗	DIV+ VRFA	0,0675∗∗∗	DIV+ VRFA
	γ_1_	0,0027		0,0092		−0,0004		−0,0042	
	γ_2_	−0,0086∗		0,0150		−0,0022		0,0152	
	γ_3_	0,0031		−0,0072		0,0026		0,0077	
**AI INDEX**	γ_0_	0,3820∗∗∗	VRFA	0,2056∗∗∗	VRFA	0,0962∗∗∗	DIV+ VRFA or VRFO	0,1902∗∗∗	VRFA
	γ_1_	0,0006		0,0166		−0,0001		0,0035	
	γ_2_	−0,0080		0,0046		0,0039∗		0,0186	
	γ_3_	0,0026		0,0179		−0,0038∗∗		0,0225	

Notes: The table displays the estimation results for various financial assets. ‘VRFA’ stands for low safe-haven value, indicating a weak safe-haven characteristic. ‘VRFO’ denotes a strong safe-haven value, signifying a robust safe-haven attribute. ‘DIV’ refers to an asset's role as a Diversifier. The symbols ∗∗∗, ∗∗, and ∗ indicate significance levels at 1 %, 5 %, and 10 %, respectively.

During the conflict between Russia and Ukraine, Bitcoin played a variable role as a safe haven asset. It was a weak safe haven for ATS at the 5 % quantile but exhibited strong safe haven properties for PRO at the same quantile. This aligns with the findings of researchers like Kayral et al. [83] and Abdullah et al. [84], who noted Bitcoin's effectiveness as a safe haven during geopolitical upheavals. However, gold's performance diverged from its traditional role as a consistent safe haven during this particular crisis, contradicting views from researchers such as Fakhfekh et al. [27] and Abdullah et al. [84], who maintained that gold remains a steadfast safe haven during such conflicts. Additionally, Huynh et al. [75] explored the role of AI and robotics stocks in portfolio diversification during economic instability, highlighting Bitcoin and gold as key assets for hedging, particularly in times of economic downturns.

In the case of gold-backed cryptocurrencies, the pattern differed. DGX emerged as a strong safe haven for SYM at both the 5 % and 10 % quantiles. It also proved to be a strong safe haven for the AI INDEX at the 1 % quantile. PAXG, on the other hand, was a strong safe haven solely for HLX at the 1 % level during the conflict.

This variation in the safe haven abilities of these assets during the crisis points to a complex interaction of investor perceptions, market conditions, and the unique attributes of each asset. For example, the varying effectiveness of Bitcoin as a safe haven across different quantiles may reflect its changing acceptance within the investor community under specific conditions. Similarly, the strong performance of gold-backed cryptocurrencies in certain instances might signal increasing investor trust in the fusion of traditional and digital safe haven assets. This analysis offers valuable insights into the performance of these assets during the Russian-Ukrainian crisis and provides a nuanced perspective that can aid in shaping investment strategies and risk management approaches. As the financial landscape evolves with the blending of cryptocurrencies and traditional assets, such data will be essential for stakeholders to navigate future crises effectively.

**Fig. 2:**
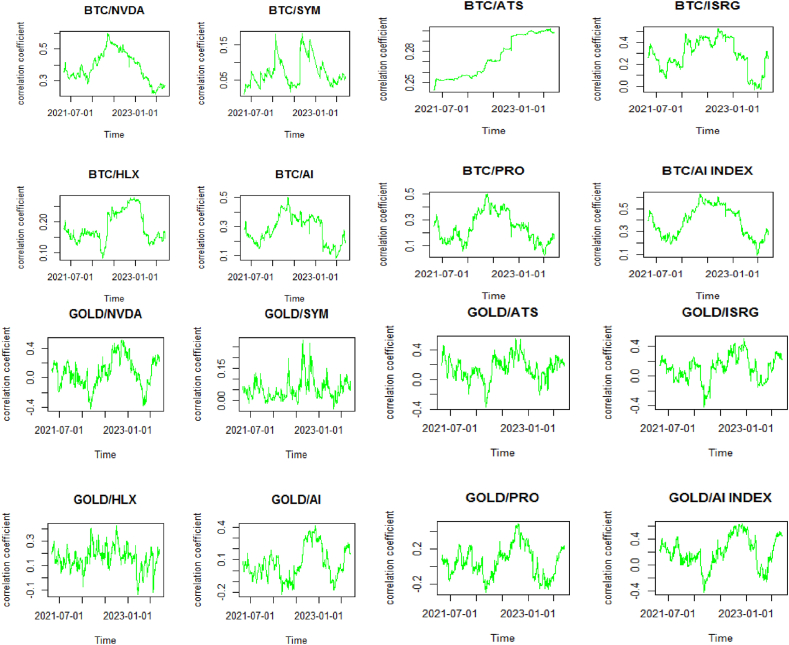

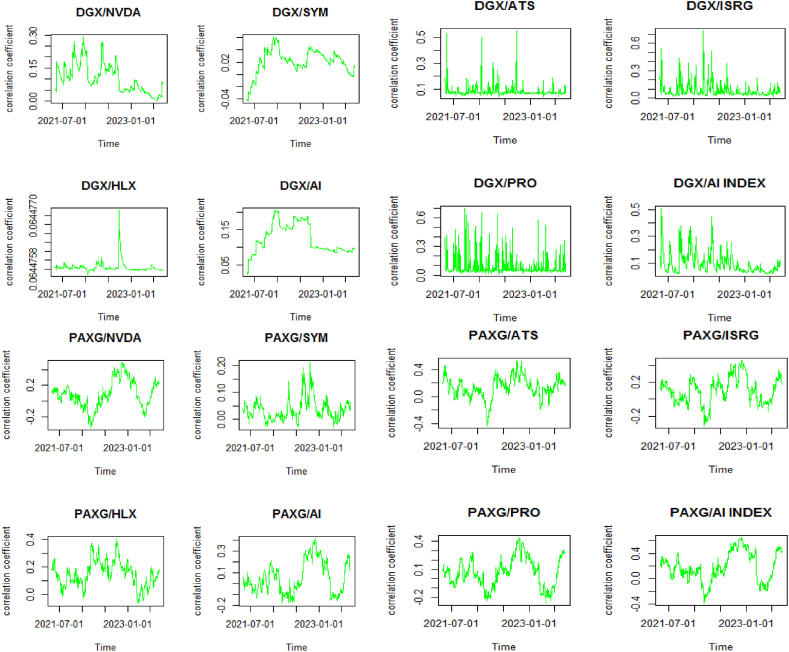
Correlation evolution series

These results are confirmed by Fig. 2, which illustrates how Bitcoin and DGX have been considered assets for diversification, playing a role similar to that of gold, which is traditionally recognized as a safe haven by investors. Particularly, investors holding stocks in companies specializing in AI—apart from the notable exception of SYM—have leaned on these digital and physical assets during the initial phases of the Ukrainian crisis and the banking crisis of 2023. Gold has been particularly effective as a hedge against the risks associated with the stocks of companies like AI and ISRG, providing a layer of protection amidst economic turbulence. Furthermore, PAXG has mirrored gold's function as a financial protector and has likewise been adopted as a safe haven by investors with stakes in the AI sector, excluding SYM and HLX, especially at the outset of the Ukrainian crisis. At the beginning of the 2023 banking crisis, PAXG also demonstrated its role as a safe haven for these investors, except for those with shares in SYM. PAXG, akin to gold, is perceived as a hedge for investors in companies like AI and ISRG and as a diversification tool for others, underscoring its strategic significance in managing portfolios to navigate through diverse economic crises. Our results align with the findings of Choudhury et al. [85], Akhtaruzzaman et al. [60], Ji et al. [61], and Díaz et al. [33].

### 5.3 Hedging properties during crises

The results of the two regression models, detailed in Equation (13), are comprehensively displayed in Table 4. These findings indicate a notable shift in the function of the same instrument across most series during the two distinct crises: the Russian-Ukrainian conflict and the SVB crisis.

**Table 4:** Estimation results of dummy variable equation.

Russian-Ukrainian conflict				SVB crisis			
	Coef.	Estimate	Role		Coef.	Estimate	Role
**BTC/NVDA**	β_0_	0,3879∗∗∗	VRFA	**BTC/NVDA**	β_0_	0,2634∗∗∗	–
	β_1_	0,0091			β_1_	0,0577∗∗∗	
**BTC/SYM**	β_0_	0,0710∗∗∗	DIV	**BTC/SYM**	β_0_	0,0505∗∗∗	DIV
	β_1_	0,0264∗∗∗			β_1_	0,0304∗∗∗	
**BTC/HLX**	β_0_	0,1856∗∗∗	VRFO	**BTC/HLX**	β_0_	0,1473∗∗∗	–
	β_1_	−0,0353∗∗∗			β_1_	0,0282∗∗∗	
**BTC/AI**	β_0_	0,2761∗∗∗	–	**BTC/AI**	β_0_	0,1578∗∗∗	–
	β_1_	0,0261∗			β_1_	0,0027	
**BTC/ATS**	β_0_	0,2749∗∗∗	VRFA	**BTC/ATS**	β_0_	0,2994∗∗∗	VRFA
	β_1_	−0,0002			β_1_	−0,0002	
**BTC/ISRG**	β_0_	0,2827∗∗∗	–	**BTC/ISRG**	β_0_	0,0975∗∗∗	DIV
	β_1_	0,0379∗			β_1_	0,1611∗∗∗	
**BTC/PRO**	β_0_	0,2348∗∗∗	–	**BTC/PRO**	β_0_	0,1210∗∗∗	–
	β_1_	0,0455∗∗∗			β_1_	0,0593∗∗∗	
**BTC/AI INDEX**	β_0_	0,3845∗∗∗	VRFA	**BTC/AI INDEX**	β_0_	0,2289∗∗∗	–
	β_1_	−0,0061			β_1_	0,0578∗∗∗	
**GOLD/NVDA**	β_0_	0,0941∗∗∗	DIV + VRFO	**GOLD/NVDA**	β_0_	0,0057	VRFA
	β_1_	−0,2659∗∗∗			β_1_	0,0189	
**GOLD/SYM**	β_0_	0,0556∗∗∗	DIV + VRFO	**GOLD/SYM**	β_0_	0,0477∗∗∗	DIV
	β_1_	−0,0133∗			β_1_	0,0280∗∗∗	
**GOLD/HLX**	β_0_	0,1668∗∗∗	VRFA	**GOLD/HLX**	β_0_	0,1158∗∗∗	VRFO
	β_1_	−0,0012			β_1_	−0,1125∗∗∗	
**GOLD/AI**	β_0_	0,0720∗∗∗	DIV + VRFO	**GOLD/AI**	β_0_	0,0332∗∗∗	DIV
	β_1_	−0,0867∗∗∗			β_1_	0,0571∗	
**GOLD/ATS**	β_0_	0,1726∗∗∗	VRFO	**GOLD/ATS**	β_0_	0,1584∗∗∗	VRFO
	β_1_	−0,2999∗∗∗			β_1_	−0,1959∗∗∗	
**GOLD/ISRG**	β_0_	0,1324∗∗∗	VRFO	**GOLD/ISRG**	β_0_	0,1002∗∗∗	VRFO
	β_1_	−0,2502∗∗∗			β_1_	−0,0948∗∗	
**GOLD/PRO**	β_0_	0,0695∗∗∗	DIV + VRFO	**GOLD/PRO**	β_0_	−0,0368∗∗∗	COUV + VRFO
	β_1_	−0,2261∗∗∗			β_1_	−0,0859∗∗	
**GOLD/AI INDEX**	β_0_	0,2271∗∗∗	VRFO	**GOLD/AI INDEX**	β_0_	0,1434∗∗∗	VRFA
	β_1_	−0,3331∗∗∗			β_1_	−0,0839	
**DGX/NVDA**	β_0_	0,1061∗∗∗	VRFO	**DGX/NVDA**	β_0_	0,0285∗∗∗	DIV
	β_1_	−0,0326∗∗∗			β_1_	0,0240∗∗∗	
**DGX/SYM**	β_0_	0,0208∗∗∗	DIV	**DGX/SYM**	β_0_	0,0156∗∗∗	DIV
	β_1_	0,0039			β_1_	0,0096∗∗∗	
**DGX/HLX**	β_0_	0,0645∗∗∗	DIV + VRFO	**DGX/HLX**	β_0_	0,0645∗∗∗	DIV + VRFO
	β_1_	0,0000∗∗∗			β_1_	0,0000∗∗	
**DGX/AI**	β_0_	0,1236∗∗∗	VRFA	**DGX/AI**	β_0_	0,0921∗∗∗	DIV + VRFA
	β_1_	0,0057			β_1_	−0,0012	
**DGX/ATS**	β_0_	0,0849∗∗∗	DIV + VRFA	**DGX/ATS**	β_0_	0,0764∗∗∗	DIV + VRFA
	β_1_	−0,0031			β_1_	−0,0026	
**DGX/ISRG**	β_0_	0,0811∗∗∗	DIV	**DGX/ISRG**	β_0_	0,0586∗∗∗	DIV
	β_1_	0,0632∗∗∗			β_1_	0,0151∗	
**DGX/PRO**	β_0_	0,0829∗∗∗	DIV + VRFA	**DGX/PRO**	β_0_	0,0734∗∗∗	DIV + VRFA
	β_1_	−0,0086			β_1_	−0,0172	
**DGX/AI INDEX**	β_0_	0,0994∗∗∗	DIV + VRFO	**DGX/AI INDEX**	β_0_	0,0488∗∗∗	DIV + VRFA
	β_1_	−0,0219∗			β_1_	0,0073	
**PAXG/NVDA**	β_0_	0,1095∗∗∗	VRFO	**PAXG/NVDA**	β_0_	0,0360∗∗	DIV + VRFA
	β_1_	−0,2413∗∗∗			β_1_	0,0397	
**PAXG/SYM**	β_0_	0,0378∗∗∗	DIV + VRFO	**PAXG/SYM**	β_0_	0,0255∗∗∗	DIV + VRFA
	β_1_	−0,0147∗∗			β_1_	0,0180∗∗∗	
**PAXG/HLX**	β_0_	0,1468∗∗∗	–	**PAXG/HLX**	β_0_	0,0779∗∗∗	DIV + VRFO
	β_1_	0,0089			β_1_	−0,0623∗∗∗	
**PAXG/AI**	β_0_	0,0623∗∗∗	DIV + VRFO	**PAXG/AI**	β_0_	−0,0056	COUVFA
	β_1_	−0,0563∗∗			β_1_	0,1233∗∗∗	
**PAXG/ATS**	β_0_	0,1619∗∗∗	VRFO	**PAXG/ATS**	β_0_	0,1462∗∗∗	VRFO
	β_1_	−0,3210∗∗∗			β_1_	−0,1829∗∗∗	
**PAXG/ISRG**	β_0_	0,1182∗∗∗	VRFO	**PAXG/ISRG**	β_0_	0,0619∗∗∗	DIV + VRFO
	β_1_	−0,2090∗∗∗			β_1_	−0,0547∗	
**PAXG/PRO**	β_0_	0,0807∗∗∗	DIV + VRFO	**PAXG/PRO**	β_0_	−0,0062	COUV + VRFA
	β_1_	−0,2068∗∗∗			β_1_	−0,0599	
**PAXG/AI INDEX**	β_0_	0,2080∗∗∗	VRFO	**PAXG/AI INDEX**	β_0_	0,1147∗∗∗	VRFA
	β_1_	−0,3043∗∗∗			β_1_	−0,0505	

Notes: The table provides the results of our estimations. ‘VRFA’ represents assets with a low safe-haven value, while ‘VRFO’ indicates assets with a strong safe-haven value. ‘DIV’ is used to denote assets that function as Diversifiers. ‘COUVFA’ is used to denote low hedging asset. The symbols ∗∗∗, ∗∗, and ∗ denote significance at the 1 %, 5 %, and 10 % levels, respectively.

Gold's performance, traditionally regarded as a robust safe haven, maintained its characteristic role, except for HLX, which became a weaker safe haven during the Russian-Ukrainian conflict. During the SVB Collapse crises, gold's role evolved, becoming a significant safe haven for stocks like HLX, ATS, ISRG, and PRO, underscoring its conventional status as a reliable store of value during economic uncertainties. Conversely, for stocks like SYM and AI, gold functioned more as a risk diversifier than a safe haven during the crisis, indicating that while it offered some risk reduction, it didn't entirely shield investors from the impacts of the crisis. This could reflect a nuanced understanding of gold's role or specific characteristics of these sectors. Our results are consistent with the findings of Baur and Lucey [82], Beckmann et al. [86], Gözde and Ünalmış (2014), Dutta et al. [62], Salisu et al. [63], Azmi [65], Jin and Tian [57], and Ming et al. [87].

These findings are vital for portfolio management, particularly in formulating strategies to mitigate the impacts of crises. They suggest that both Becket-gold and Bitcoin act as effective diversifiers across various sectors and during economic upheavals for most of the series examined. Gold, on the other hand, appears to be a more situational asset, with its role as a safe haven or diversifier changing according to the specific crisis and the stocks in question. The unique functions of Bitcoin, Becket-gold, and gold across different technology and AI stocks highlight the necessity of strategic asset selection when constructing a crisis-resistant portfolio. Investors and portfolio managers are advised to consider the timing, nature of financial crises, and sector-specific dynamics in their decision-making processes, as guided by these empirical findings.

## 6 Conclusion

This article evaluates the hedging, diversification, and safe-haven attributes of gold, Bitcoin, and gold-backed stablecoins against the backdrop of major AI companies and stocks during political and banking crises. Our analysis, using data from April 30, 2021, to September 15, 2023, employs the ADCC model integrated with four GARCH family models and two residual distributions, resulting in a total of eight comprehensive models.

Our findings indicate nuanced asset behaviors under different crisis conditions. During the Russian-Ukrainian crisis, Bitcoin showed limited safe-haven properties for ATS and stronger ones for PRO. Gold-backed cryptocurrencies, particularly DGX, emerged as significant safe havens for SYM and the AI INDEX. PAXG demonstrated strong safe-haven qualities for HLX during this period. Notably, Bitcoin was a key risk diversifier for all technology and AI stocks in the lead-up to and during the SVB banking crisis. In contrast, gold was a consistent diversifier for all studied stocks before the SVB crisis, except for PRO, where it acted more as a hedging tool. During the banking crisis, gold was a solid safe haven for HLX, ATS, ISRG, and PRO, and a diversifier for SYM and AI. In fact, gold and the gold-backed cryptocurrency DGX emerge as the most efficient safe haven assets against AI company stocks when compared to Bitcoin, providing robust support for investors seeking stability in uncertain market conditions. This finding underscores the enduring appeal and resilience of gold, a traditional safe haven asset, while also highlighting the potential of newer assets like DGX to fulfill a similar role.

The empirical findings of the study provide significant insights for individuals involved in financial markets, underscoring the necessity of comprehending the relationships between assets to manage risks efficiently. This emphasizes the importance of tailoring portfolio allocations to suit various risk levels, especially during times of heightened political tension or banking crises. Such customization becomes crucial in navigating through turbulent market conditions, where the ability to adjust asset allocations strategically can help mitigate potential risks and optimize investment returns. These insights are particularly valuable for financial advisors, who are responsible for guiding clients through complex market dynamics. By understanding the intricate behaviors of assets, such as those of AI companies and safe-haven assets like gold, during uncertain periods, financial advisors can make informed decisions, thus safeguarding their clients' investments and ensuring effective risk management strategies are in place. Overall, this information equips financial advisors with the knowledge needed to make prudent decisions in turbulent times, ultimately helping investors navigate through uncertain market environments more effectively.

As we conclude this study, it's crucial to recognize its limitations and identify directions for future research. The current methodology, employing the ADCC model alongside GARCH family models, is robust but may not capture the full intricacies of asset relationships during crises. Future studies could explore diverse econometric approaches, including machine learning algorithms and advanced time-series models, to gain deeper insights into the non-linear dynamics of financial markets. The development of hybrid models that merge established econometric practices with modern computational techniques could also offer a more detailed perspective on market behavior during volatile times. Additionally, future research could involve dynamic hedging analyses for commodities and cryptocurrencies, tailored to different AI and Fintech companies, across various crisis contexts. Such investigations would be invaluable in enhancing our understanding and management of risks within the dynamic sphere of financial markets.

## CRediT authorship contribution statement

**Wael Dammak:** Writing – review & editing, Supervision. **Mohamed Fakhfekh:** Writing – original draft, Validation, Formal analysis, Conceptualization. **Hind Alnafisah:** Validation, Funding acquisition. **Ahmed Jeribi:** Software, Resources, Methodology, Data curation.

## Declaration of competing interest

The authors declare that they have no known competing financial interests or personal relationships that could have appeared to influence the work reported in this paper.

## Figures and Tables

**Table:** 

Variable	Definition
**NVDA**	NVIDIA Corporation is an American technology company specializing in the design of graphics processing units (GPUs) and related technologies for artificial intelligence (AI), machine learning (ML), and virtual reality (VR).
**SYM**	Symbotic Inc. is a company specializing in automation technology for warehouses and supply chains. It develops robotic systems to enhance efficiency and accuracy in logistics operations.
**HLX**	Helix Energy Solutions Group, Inc. is an American company specializing in offshore services for the oil and gas industry.
**AI**	C3.ai, Inc. is a software company specializing in providing AI and machine learning solutions for various industries, including energy, healthcare, and finance.
**ATS**	ATS Corporation is a technology company specializing in providing information and technology management solutions for governments and businesses.
**ISRG**	Intuitive Surgical, Inc. is an American company leading in the field of robotic surgery. It is known for its da Vinci Surgical System, a robotic system for minimally invasive surgery.
**PRO**	PROS Holdings, Inc. is a software company that provides pricing and sales optimization solutions for businesses, using AI and machine learning technologies.
**AI Index**	The AI Index is an indicator that tracks and measures the evolution and impact of artificial intelligence across various sectors of the economy, including research, industry, education, and society.
**Bitcoin**	Bitcoin is a decentralized digital currency created in 2009. It is based on blockchain technology and is used as a medium of exchange and store of value.
**Gold**	Gold is a precious metal used as a store of value, medium of exchange, and material for jewelry and art objects.
**DGX**	Digix Gold (DGX) is a gold-backed cryptocurrency where each DGX represents 1 g of physical gold stored in secure vaults.
**PAXG**	Paxos Gold (PAXG) is another gold-backed cryptocurrency where each PAXG represents 1 troy ounce of physical gold stored in secure facilities.

## Footnotes

1 https://kpmg.com/xx/en/home/insights/2022/09/kpmg-global-tech-report-2022.html.

2 https://www.nerdwallet.com/article/investing/ai-stocks-invest-in-artificial-intelligence.
